# “Associated” or “Secondary” IgA nephropathy? An outcome analysis

**DOI:** 10.1371/journal.pone.0221014

**Published:** 2019-08-09

**Authors:** Bogdan Obrișcă, Gabriel Ștefan, Mihaela Gherghiceanu, Eugen Mandache, Gener Ismail, Simona Stancu, Bianca Boitan, Oana Ion, Gabriel Mircescu

**Affiliations:** 1 Nephrology Department, Fundeni Clinical Institute, Bucharest, Romania; 2 Nephrology Department,”Carol Davila” University of Medicine and Pharmacy, Bucharest, Romania; 3 ”Dr. Carol Davila” Teaching Hospital of Nephrology, Bucharest, Romania; 4 ”Victor Babes” National Institute of Pathology, Bucharest, Romania; International University of Health and Welfare, School of Medicine, JAPAN

## Abstract

**Background:**

Whether differences in outcome between primary (pIgAN) and secondary IgA nephropathy (sIgAN) exist is uncertain.

**Methods:**

We conducted a retrospective, observational study that included all histologically diagnosed IgAN patients between 2010–2017 (N = 306), 248 with pIgAN and 58 with sIgAN. To obtain samples with similar risk of progression, sIgAN patients were grouped as liver disease and autoimmune/viral disease and propensity score matched to corresponding pIgAN samples. Univariate (Kaplan Meier) and multivariate time-dependent (Cox modelling) analyses were performed to identify predictors of the composite end-point (doubling of serum creatinine, end-stage kidney disease or death).

**Results:**

Of the whole cohort, 20% had sIgAN (6% alcoholic cirrhosis, 6% autoimmune disease and 8% viral infections). sIgAN patients were older, had more comorbidities, lower proteinuria and higher haematuria, but similar distribution in MESTC lesions and eGFR as those with pIgAN. They reached the end-point in similar proportions with those with pIgAN (43 vs. 30%; p = 0.09) but their mortality was higher (19 vs. 3%; p<0.0001). Both in unmatched (HR 0.80, 95%CI 0.42–1.52; p = 0.5) and matched samples (log-rank test: liver disease-IgAN vs. pIgAN, p = 0.1; autoimmune/viral-IgAN vs. pIgAN, p = 0.3), sIgAN was not predictive for end-point. In analyses restricted only to sIgAN, those with viral infections (HR, 10.98; 95% CI, 1.12–107.41; p = 0.03) and lower eGFR (HR, 0.94; 95%CI, 0.89–0.98; p = 0.007) had a worse prognosis. Immunosuppression did not influence outcome.

**Conclusions:**

The differences in MESTC score and outcome between pIgAN and sIgAN seems to be minimal, suggesting that “associated” describes better than “secondary” the relationship among the two. Immunosuppression did not to influence outcome of sIgAN.

## Introduction

Immunoglobulin A nephropathy (IgAN) is the commonest glomerulonephritis diagnosed by kidney biopsy globally (Europe 23%; Asia 39%) and an important cause of end-stage renal disease (ESRD)[1,2]. IgAN is an autoimmune disorder with renal-limited or systemic manifestations (IgA vasculitis or Henoch-Schönlein purpura), determined by a chain of pathogenic events that translates the production of autoantibodies against circulating galactose-deficient IgA1 into subsequent glomerular and tubulointerstitial injury[3,4].

However, IgAN was histopathologically diagnosed with variable frequency in association with several conditions[5–16]. In some of these conditions, IgAN may be pathogenetically related to the underlying condition (cirrhosis, HBV infection, inflammatory bowel disease, spondylarthritis, Hashimoto’s thyroiditis, psoriasis), while in others the association may be coincidental (non-Hodgkin and Hodgkin lymphoma)[6,8, 9, 12]. As whether these associations are due to a common pathogenesis or are simply coincidental is largely unknown[8], IgAN diagnosed by biopsy in patients with another condition is labeled either as “secondary”, when a common pathogenesis is presumed, or as “associated”, when a coincidental occurrence is presumed. In this paper we will use the term “secondary IgAN (sIgAN)” in both situations.

Information on the clinical phenotype, morphologic lesions, outcome and treatment response of sIgAN is scarce and come from small cohorts or series of cases, few of them including comparisons with primary IgAN (pIgAN)[5–16]. Accordingly, to date there are no features to accurately discriminate primary from secondary IgAN.

The Oxford Classification of IgAN has been shown to be related to renal outcome in patients with primary IgAN[17,18]. However, the patients with sIgAN have been excluded from validation studies and whether this classification can be applied to this subset of patients is currently unknown.

Moreover, the prognosis of patients with primary IgAN is extremely variable and the risk of progression may be influenced by regional environmental factors, differences in racial composition and genetic susceptibility[19]. The 10-year renal survival rate is reported to be between 60% and 90%, while up to 50% may reach end-stage renal disease (ESRD) within 20 years of diagnosis[20,21]. However, if secondary IgAN has a different outcome from primary IgAN has not been systematically addressed. The secondary IgAN is extremely heterogenous and the prognosis of patients with secondary IgAN may be influenced by the underlying condition and their outcome may be difficult to be directly compared to that of patients with primary IgAN.

To overcome these limitations, we conducted a retrospective, observational study intending to describe the clinical phenotype and the outcome in patients with secondary IgAN as compared to primary IgAN, using propensity score matching to obtain samples with a similar risk of progression.

## Materials and methods

### Patient selection

This is a retrospective, observational study addressing the outcome of patients with IgAN diagnosed in two tertiary centers.

**Fig 1:**
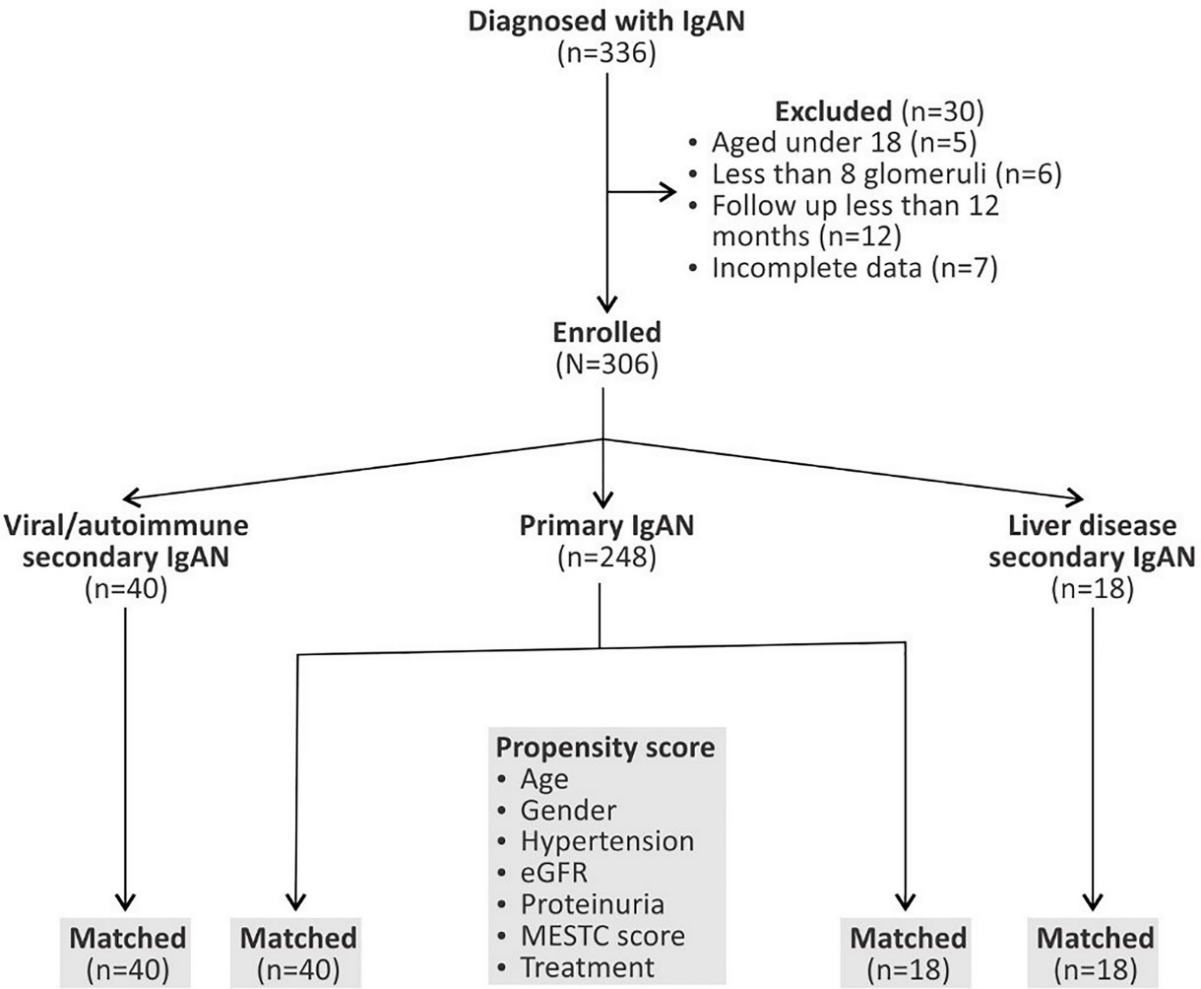
Patients’ flow chart and the formation of samples used for matching.

All patients with histologically proved IgAN between 01.01.2010 and 12.31.2017 (n = 336) were considered for inclusion. Those with ages under 18, those whose renal biopsy specimen contained less than 8 scorable glomeruli, with insufficient clinical data or with a shorter than 12 months follow-up at 06.01.2018 were excluded from the analysis, leaving a final cohort of 306 patients (**Fig 1**).

The diagnosis of IgAN was based on light microscopy, immunofluorescence (dominant IgA in the mesangium) and electron-microscopy (para-mesangial electron-dense deposits). All kidney biopsies were reviewed and scored according to the 2016 revised Oxford Classification[18] by two independent pathologists.

All patients underwent a systematic screening for disorders reported to be associated with IgAN, and thereafter classified as having primary IgAN (n = 248) or secondary IgAN (n = 58)[8,22].

The conditions associated with IgAN in this cohort were: liver diseases (alcoholic cirrhosis), viral infections (hepatitis B virus and hepatitis C virus) and autoimmune disorders (psoriasis, ankylosing spondylitis, rheumatoid arthritis, autoimmune thyroiditis). The patients with secondary IgAN were further grouped as liver-disease and autoimmune/viral secondary IgAN (**Fig 1**, **Table 1**).

**Table 1:** Conditions associated with IgA nephropathy in the study cohort (N = 306).

Category	Group		Associated disease
**Primary IgAN** n = 248 (80%)	-	-	-
**Secondary IgAN** n = 58 (20%)	**Liver disease** n = 18 (6%)		Alcoholic cirrhosis n = 18 (6%)
	**Autoimmune/Viral** n = 40 (14%)	**Autoimmune disorders** n = 17 (6%)	Ankylosing spondylitis n = 6 (2%)
			Rheumatoid arthritis n = 3 (1%)
			Psoriasis n = 7 (2%)
			Thyroiditis 1 (1%)
		**Viral infection** n = 23 (8%)	Hepatitis B virus n = 17 (6%)
			Hepatitis C virus n = 6 (2%)

### Clinical and histological parameters

The clinical variables obtained by reviewing the patient’s medical records at the time of kidney biopsy were age, gender, Charlson comorbidity index[23], obesity (defined as a body mass index over 30 kg/m^2^), diabetes mellitus and arterial hypertension (defined as blood pressure over 140/90 mmHg or use of antihypertensive agents), therapy with renin-angiotensin-aldosterone system inhibitors (RASI) and immunosuppressive medication (IS).

Laboratory data included serum creatinine, estimated glomerular filtration rate (eGFR, calculated by CKD-EPI equation), serum albumin, proteinuria (g/g creatinine) and haematuria (cells/mmc).

### Study endpoints

The primary study composite endpoint was defined as doubling of serum creatinine, ESRD (dialysis or renal transplant) or death, whichever came first.

### Statistical analysis

Continuous variables were expressed as either mean or median and 95% confidence interval (95%CI) and categorical variables as percentages. Differences between groups were assessed in case of continuous variables by Student *t* test or by Mann-Whitney test, according to their distribution, and in case of categorical variables by Pearson χ^2^ test.

The probability of event-free survival was assessed by Kaplan-Meyer method and the log-rank test was used for comparisons.

Univariate and multivariate (Cox proportional hazard ratio) analyses were performed to identify independent predictors of the endpoint. The results of Cox analyses are expressed as a hazard ratio (HR) and 95% confidence interval (95% CI).

Given the retrospective nature of this study, patients with primary and secondary IgAN could differ in baseline characteristics and comorbidities, which might confound the outcome. Therefore, in the first step we compared clinical and pathological features, treatment and outcome of pIgAN and sIgAN in the entire cohort. Thereafter, to obtain samples with similar risk of progression, we used a propensity score to match groups of secondary IgAN patients, i.e. liver disease and autoimmune/viral, to patients with primary IgAN. The variables used to compute the propensity score were age, gender, hypertension, eGFR, proteinuria, MESTC score and treatment. We matched patients with the closest propensity score with a maximum difference of ±0.5.

Finally, comparisons were made between patients with secondary IgAN, according to the underlying condition, i.e. viral, autoimmune and liver disease.

In all analyses, *p* values are two-tailed and all *p* values less than 0.05 were considered statistically significant.

Statistical analyses were performed using the SPSS program (SPSS version 20, Chicago, IL) and XLSTAT (Addinsoft 2019, XLSTAT statistical and data analysis solution. https://www.xlstat.com. Boston, USA).

### Ethics

The study was conducted with the provisions of the Declaration of Helsinki and the protocol was approved by the local ethics committee (The Ethics Council of Fundeni Clinical Institute, Registration number: 23250). The need for informed consent was waived due to exclusive use of deidentified information and the retrospective nature of the study.

## Results

### Study cohort

The study cohort included 306 patients (71% male). At the time of kidney biopsy, their age was 43 (95%CI, 42–45) years, eGFR was 42.0 (95%CI, 38.5–46.2) mL/min per 1.73m^2^, proteinuria was 1.3 (95%CI, 1.2–1.5) g/g and 79% of patients had arterial hypertension. The median Charlson comorbidity index was 2.3 (95%, 2.1–2.5).

On histopathologic examination, mesangial hypercellularity was present in 93% of patients, endocapillary hypercellularity in 26%, segmental glomerulosclerosis in 52%, tubular atrophy and interstitial fibrosis >25% (T1+T2) in 33% and 22% showed crescents in at least one glomerulus. The median MESTC score was 2.5 (95%CI, 2.3–2.6) (**Table 2**).

**Table 2:** Baseline characteristics and outcome of the whole cohort.

	All (n = 306)	Primary IgAN (n = 248)	Secondary IgAN (n = 58)	P
Age (years)	43 (42, 45)	42 (40, 43)	55 (50, 58)	<0.001
Male gender (%)	71	69	79	0.1
Charlson comorbidity index	2.3 (2.1, 2.5)	1.9 (1.7, 2.2)	3.8 (3.2, 4.5)	<0.001
Obesity (%)	28	29	26	0.6
Diabetes mellitus (%)	7	7	10	0.3
Hypertension (%)	79	82	71	0.06
Serum creatinine (mg/dL)	1.7 (1.6, 1.9)	1.7 (1.6, 1.9)	1.8 (1.4, 2.4)	0.6
eGFR (mL/min/1.73m2)	42.0 (38.5, 46.2)	42.8 (39.0, 47.0)	35.2 (25.5, 51.2)	0.3
Proteinuria (g/g creatinine)	1.3 (1.2, 1.5)	1.4 (1.2, 1.7)	1.0 (0.7, 1.4)	0.01
Haematuria (cells/mm3)	110 (80, 180)	84 (65, 146)	190 (100, 230)	0.01
Serum albumin (g/dL)	4.1 (4.0, 4.2)	4.1 (4.0, 4.2)	4.1 (3.9, 4.2)	0.1
**Renal biopsy (%)**				
M1	93	94	91	0.5
E1	26	26	28	0.7
S1	52	53	47	0.3
T1/2	20/13	20/14	21/10	0.7
C1/2	14/7	14/8	17/3	0.4
MESTC score	2.5 (2.3, 2.6)	2.5 (2.3, 2.7)	2.3 (1.9, 2.7)	0.3
**Treatment (%)**				
Immunosuppression therapy	52	54	41	0.08
RASI	71	75	57	<0.01
**Outcome (%)**				
Double sCr	11	11	12	0.7
ESRD	15	16	12	0.4
Kidney end-point (double sCr or ESRD)	26	27	24	0.6
Death	6	3	19	<0.0001
Composite end-point (double sCr, ESRD, death)	32	30	43	0.09

***Abbreviations*:** eGFR, estimated glomerular filtration rate; sCr, serum creatinine; IgAN—IgA nephropathy; M1, mesangial hypercellularity; E1, endocapillary hypercellularity; S1, segmental glomerulosclerosis; T1/2, tubular atrophy and interstitial fibrosis >25%; C1/2, crescents in at least one glomerulus; ESRD, end-stage renal disease

More than two thirds received RASI, while half had received some form of immunosuppressive treatment during the observation period (42% only steroids, 46% steroids and cyclophosphamide, and 12% steroids and other immunosuppressors).

The median follow-up was 32.2 (95%CI, 29.5–34.9) months. Of the entire cohort, 15% developed ESRD, 11% experienced serum creatinine doubling and 6% died.

### Primary versus secondary IgAN

Patients in the sIgAN category differed at diagnosis from those with pIgAN in almost all clinical characteristics, with the notable exception of the eGFR which was similar. sIgAN patients were older, had higher Charlson index and less frequently hypertension. Moreover, they had lower proteinuria but higher haematuria (**Table 2**). The distribution in MESTC classes was alike in both categories. Patients with pIgAN were more often treated with RASI and experienced a trend to increased usage of immunosuppressive therapy.

While the composite and kidney endpoints were reached in analogous proportions in both categories, patients with secondary IgAN had a higher mortality rate (**Table 2**).

The mean event-free survival of the whole cohort was 5.7 (95%CI, 5.3–6.2) years. In univariate analysis, pIgAN patients had a better mean event-free survival, 5.9 (95%CI 5.5–6.4) vs. 4.3 (95%CI 3.5–5.2) years (p = 0.05). The crude hazard ratio for the endpoint in primary versus secondary IgAN was 0.62 (95%, CI 0.38–1.02; p = 0.06).

In the multivariate Cox proportional hazard ratio model, the independent predictors of a poorer event-free survival were male gender, lower eGFR and higher proteinuria, while RASI therapy or immunosuppression independently predicted a better outcome. IgAN category had not a significant contribution in defining the outcome (**Table 3**).

**Table 3:** Prognostic factors in the whole cohort*.

	Univariate (HR, 95%CI)	p	Adjusted (HR, 95%CI)	p
Age (years)	1.01 (1.00, 1.03)	0.02	0.98 (0.95, 1.00)	0.08
Gender, male *vs*. female	0.67 (0.40, 1.13)	0.1	1.88 (1.06, 3.33)	0.03
Charlson comorbidity score	1.24 (1.13, 1.35)	<0.001	1.03 (0.88, 1.21)	0.6
Hypertension (yes *vs*. no)	0.57 (0.31, 1.06)	0.5	1.29 (0.63, 2.62)	0.4
eGFR (mL/min/1.73m^2^)	0.95 (0.94, 0.96)	<0.001	0.95 (0.94, 0.96)	<0.001
Proteinuria (g/g creatinine)	1.13 (1.06, 1.22)	<0.001	1.15 (1.05, 1.26)	0.001
Hematuria (cells/mm^3^)	1.07 (0.93, 1.22)	0.3	1.00 (0.87, 1.15)	0.9
MESTC score	1.41 (1.22, 1.63)	<0.001	1.04 (0.87, 1.23)	0.6
IS (no *vs*. yes)	1.00 (0.65, 1.53)	0.9	1.83 (1.14, 2.96)	0.01
RASI (no *vs*. yes)	2.60 (1.69, 3.99)	<0.001	2.02 (1.24, 3.29)	<0.01
Primary vs. secondary IgAN	0.62 (0.38, 1.02)	0.06	0.80 (0.42, 1.52)	0.5

* Cox regression (composite end-point)

Abbreviations: eGFR—estimated glomerular filtration rate; IS—immunosuppression; IgAN—IgA nephropathy; RASI—Renin angiotensin system inhibitors.

### Primary versus secondary IgAN in propensity matched samples

The differences in event-free survival between secondary IgAN groups (liver disease and autoimmune/viral disease) and primary sIgAN were evaluated in ˝case-control˝ studies, using propensity score matching. The main prognostic factors were balanced in the matched samples (**S1 Table)**.

**Fig 2:**
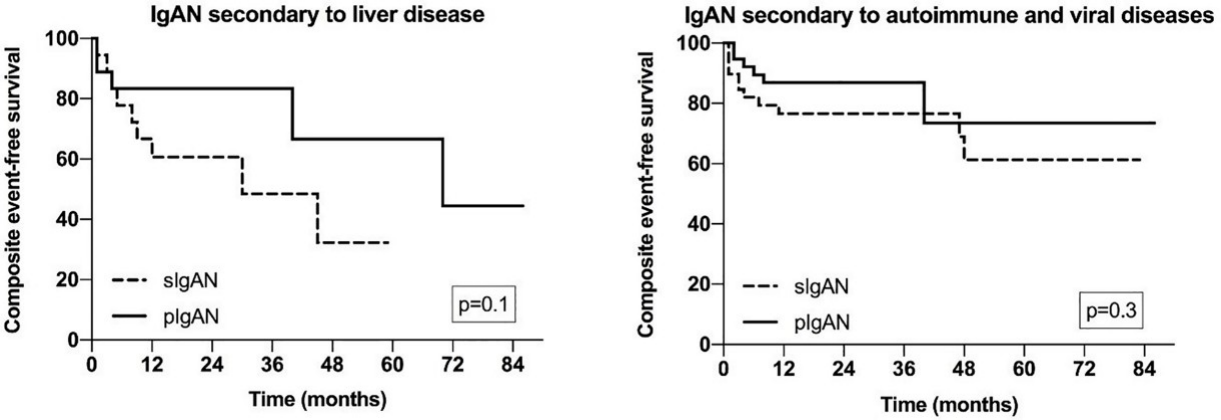
Cumulative event-free survival (Kaplan–Meier analysis) in the matched samples. Left: Liver-disease underlying IgAN vs. primary IgAN; Right: Autoimmune/viral-disease underlying IgAN vs. primary IgAN. IgAN—IgA nephropathy.

In univariate time-dependent analysis (Kaplan Meyer), the mean end-point free survival was not different in liver disease from the primary IgAN group: 2.6 (95%CI 1.7; 3.6) vs. 5.0 (95%CI 3.5; 6.5) years (p = 0.1). The same was true in case of autoimmune/viral disease group: mean end-point free survival 4.9 (95%CI 3.9; 5.9) versus 5.7 (95%CI 4.8; 6.6) years (p = 0.3) (**Fig 2**).

### IgAN underlying group of disease

**Fig 3:**
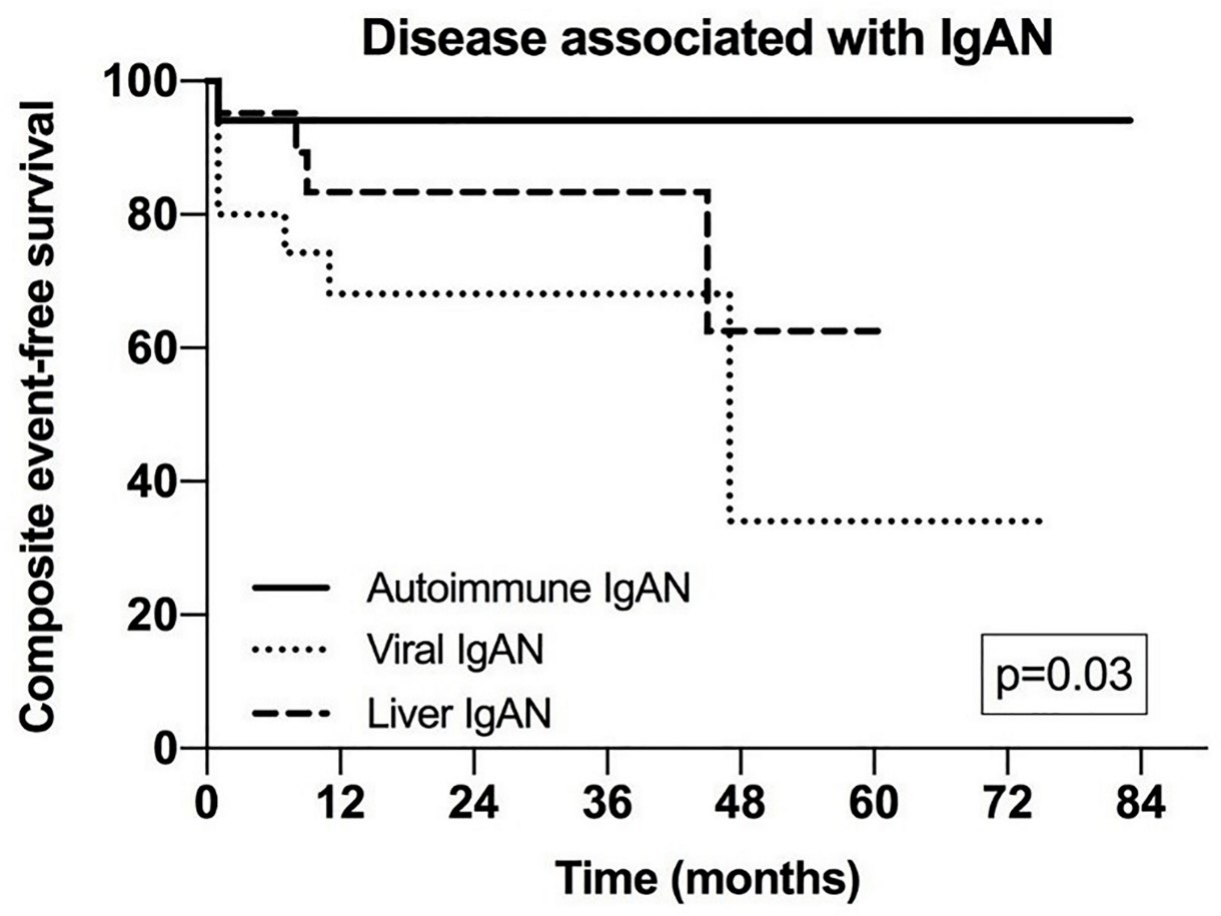
Cumulative event-free survial according to the goup of disease underlying IgA nephropathy in patients with secondary Imunoglobulin IgA nephropathy (IgAN) (Kaplan Meier analysis).

When analysing the outcome in secondary IgAN patients grouped according to underlying disease (viral infections, liver disease, autoimmune disorders), in univariate time dependent analyses, higher age, lower eGFR, higher proteinuria and MESTC score, as well as viral infection were associated with a poor event-free survival. However, in the Cox regression model, the only negative prognostic factors were a lower eGFR and the group of disease underlying IgAN, patients with viral infection having the shorter survival (**Table 4**, **Fig 3**).

**Table 4:** Prognostic factors in groups of disease underlying secondary IgA nephropathy*.

	Univariate (HR, 95%CI)	p	Adjusted Model# (HR, 95%CI)	p
Age (years)	1.07 (1.01, 1.14)	0.02	-	-
Hypertension (yes vs. no)	5.33 (0.68, 41.50)	0.1	-	-
eGFR (mL/min/1.73m2)	0.94 (0.91, 0.98)	0.005	0.94 (0.89, 0.98)	0.007
Proteinuria (g/g creatinine)	1.34 (0.98, 1.83)	0.06	1.32 (0.97, 1.79)	0.06
MESTC score	1.55 (1.09, 2.19)	0.01	-	-
IS (no vs. yes)	1.45 (0.43, 4.84)	0.5	-	-
RASI (no vs. yes)	1.68 (0.53, 5.26)	0.3	-	-
**IgAN underlying disease associated condition (vs. Autoimmune)**		0.09		0.03
• Liver disease	4.47 (0.49, 40.73)	0.1	2.33 (0.25, 21.63)	0.4
• Viral	9.36 (1.11, 78.53)	0.03	10.98 (1.12, 107.41)	0.03

* Cox regression (composite end-point)

# Backward Wald final step

Abbreviations: eGFR—estimated glomerular filtration rate; IgAN—IgA nephropathy; IS—immunosuppression; RASI—renin angiotensin system inhibitors.

**Fig 4:**
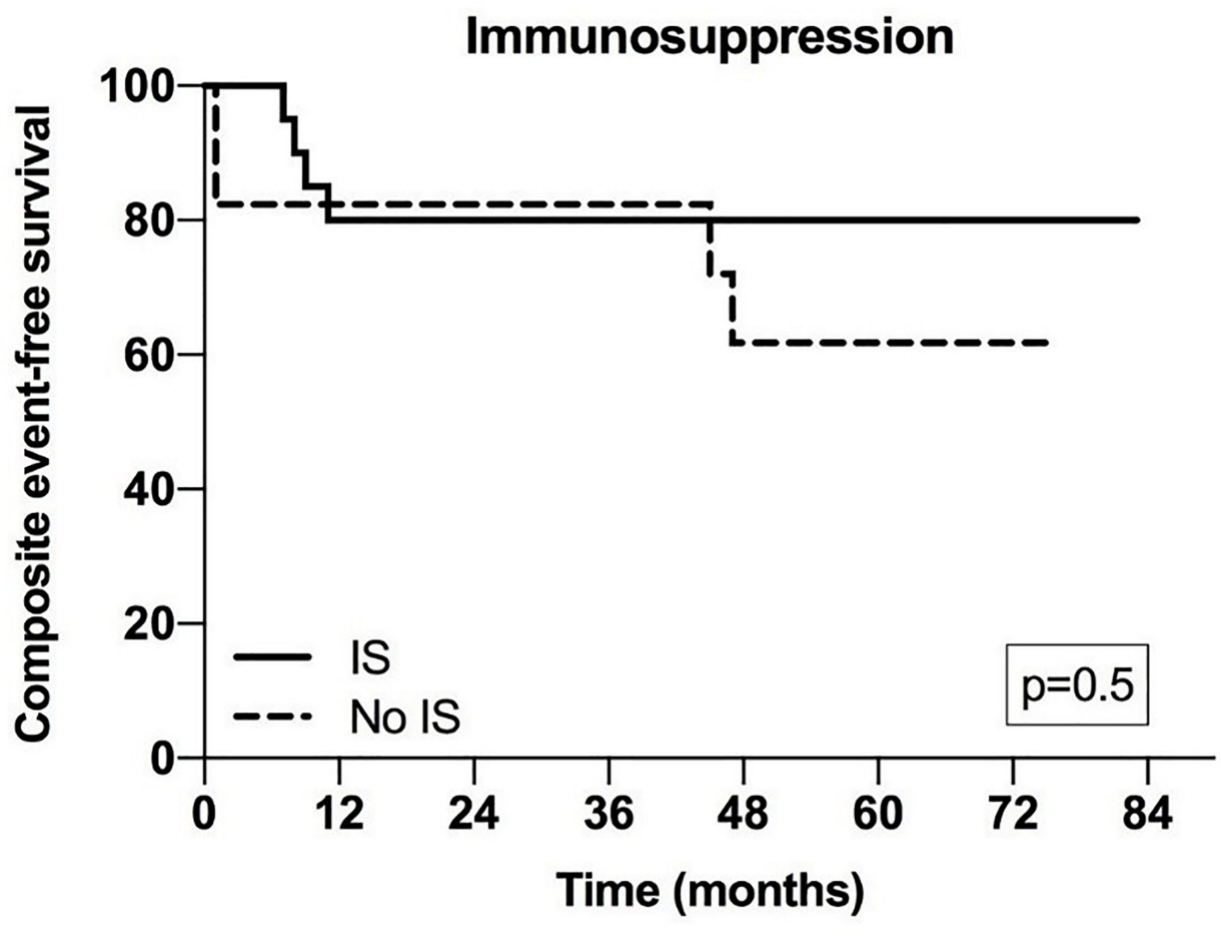
Cumulative composite event-free survial of the patients with secondary IgAN according to immunossupresion (IS) (Kaplan Meier analysis).

We then looked at the influence of the immunosuppression on outcome in patients with secondary IgAN. There were no differences at the time of IgAN diagnosis between the treatment groups regarding the known risk factors for progression: age, hypertension, eGFR, proteinuria or Oxford classification. Moreover, the event-free survival was similar (**Fig 4**, **S2 Table**).

## Discussion

In this report, the first to our knowledge to systematically evaluate the outcome between primary and secondary IgAN, there was no difference between primary and secondary IgAN, neither in the whole cohort nor in propensity score matched samples, although the clinical presentation at diagnosis was different. Moreover, the distribution according to MESTC score was similar. However, when the outcome was evaluated only in the patients with secondary IgAN, the patients with viral infection had the worst prognosis and immunosuppression did not influence the outcome.

Within our cohort of 306 consecutive patients with IgAN diagnosed by kidney biopsy, 58 (20%) concurrently had a disorder reported to be associated with IgAN[8]. These patients had mainly viral infections, liver disease or autoimmune disorders, which is in line with other reports, except a lower frequency of hepatitis B virus infection (6 vs. 20–40%)[5,13,24] and the absence of cases of chronic inflammatory bowel disease[14]. This is probably accounted for by regional epidemiologic characteristics. Thus, this cohort could be considered representative for secondary IgAN, as it covers the spectrum of diseases frequently reported to be associated with IgAN, with the notable exception of the chronic bowel inflammatory disease.

Our entire cohort differed from previous reports: older age, lower eGFR, higher proportion of high blood pressure[25]. The patients with secondary IgAN were older, had more comorbidities, lower proteinuria and higher haematuria but similar eGFR as those with primary IgAN. Accordingly, this cohort seems to have more advanced IgAN, which should be considered when interpreting outcome. However, the higher haematuria noted in patients with sIgAN might be attributed to the underlying disorders as patients with cirrhosis had more proliferative lesions on kidney biopsy (E1 in 44% of cases vs. 21% in other causes of sIgAN, S1 Table) and are more prone to a pro-hemorrhagic state (thrombocytopenia, clotting abnormalities in the setting of hepatic insufficiency).

The MESTC score, an accepted prognostic tool in IgAN, was developed and validated in patients with primary IgAN[18,25]. However, patients with secondary IgAN have been excluded from validation studies and whether MESTC score can be routinely applied to patients with secondary IgAN needs external validation in independent cohorts. Here, we report the first time in patients with secondary IgAN the scoring of lesions according to MESTC criteria[17]. Notably, there was no difference in MESTC score between primary and secondary IgAN, although the distribution in MESTC classes differed from other cohorts: M1, E1 and T1/2 were more frequent, while S1 and C were less frequent[18,25,26]. Hence, in this cohort both kidney disease clinical phenotype and histopathological features of secondary IgAN and primary IgAN are indistinctive. Although within the entire cohort we didn’t find significant differences in terms of MESTC score distribution among patients with pIgAN and sIgAN, when analyzing patients with sIgAN according to underlying comorbidity we noted that those with alcoholic cirrhosis sIgAN had more active and chronic lesions than those with autoimmune/viral sIgAN (E1, 44% vs. 21%; S1, 67% vs. 36%, T1/2, 34% vs. 29%). These findings might explain the underlying differences in outcome between patients with sIgAN.

In the whole cohort, the risk of reaching the composite end-point was not related to IgAN category (secondary vs. primary) and the independent predictors of a better outcome were the generally accepted ones—female gender, higher eGFR, lower proteinuria and therapy with RASI or immunosuppression. Thus, the outcome of patients with secondary and primary IgAN is similar. The MESTC score was not related to outcome in the fully adjusted model, which further highlights the similarity between secondary and primary IgAN. However, mortality was included in the composite end-point and the mortality rate was higher in secondary IgAN, emphasizing the importance of underlying comorbidities. Therefore, baseline differences in comorbidities may confound the observed outcome. In addition, although mortality was not included as a primary outcome in retrospective studies of pIgAN (including Oxford Classification validation studies [25]), a recent report identified an increased mortality of patients with IgAN when compared with matched controls and a 6-year life expectancy reduction[27]. Additionally, given our observation of the higher mortality rate in patients with sIgAN and in order to increase the number of events, we added mortality to the primary composite outcome. Moreover, in a recent report, Sevillano *et al* also included mortality in composite primary endpoint in a study that addressed the outcome of older patients with IgAN[28].

In order to balance the baseline characteristics, secondary IgAN patients were matched to patients with primary IgAN in two models: hepatic cirrhosis and autoimmune/viral disorders. A similar approach was undertaken by Oh *et al*[29] in a study that addressed the outcome of patients with Henoch-Schonlein purpura by comparison to patients with pIgAN.In the matched samples, outcome of secondary and primary IgAN was analogous with the exception of patients with cirrhosis that had a tendency towards a worse survival. However, in a further analysis addressing only the secondary IgAN, patients within autoimmune group had the lowest risk to reach the composite end-point, while those with viral infection, the highest. Hence, the differences in outcome between secondary and primary IgAN in this cohort seems minimal and restricted to the viral infection group.

It is debatable whether this difference in outcome requires a change in patient management. It is generally agreed that the management of patients with secondary IgAN should be directed towards the underlying disorders[8], but some patients also receive immunosuppressive agents in addition to conservative treatment[13,15]. Whether some subsets of patients can benefit from additional immunosuppression remains unknown. In our entire cohort, 52% had received at various time points immunosuppressive therapy (54% and 41% of patients with primary and secondary IgAN, respectively). In multivariate analysis, patients who received RAS inhibitors or immunosuppression showed a better outcome. However, the statistical significance was lost when restricting the analysis to secondary IgAN group. Although the small-sized cohort prevents firm conclusions, immunosuppression may be considered in patients with a rapidly progressive clinical course or extensive crescent formation on kidney biopsy. Nevertheless, the decision on initiation of immunosuppression should be well balanced against the important risk of side effects in these patients[15].

There are several limitations of this study. First, this is a retrospective study and the findings may be influenced by other variables not considered. However, this is the first study attempting to adjust the baseline differences between patients with primary and secondary IgAN by using propensity score matching. Second, the small number of patients with secondary IgAN (and of events) may prevent strong conclusions, not entirely avoided by matching. Third, this cohort has some characteristics which hamper the generalization of results: no patient with secondary IgAN to chronic inflammatory bowel disease (a frequently reported association with IgAN) was included, more advanced IgAN—suggested by lower eGFR, higher proteinuria, distinct distribution of MEST lesions—and by the higher risk of progression as compared with other reports despite of a shorter follow-up.

In conclusion, the differences in MESTC lesions and outcome between primary and secondary IgA nephropathy seems minimal and probably restricted to certain associated diseases, suggesting that “associated” describes better than “secondary” the relationship among them. Immunosuppression seems not effective in secondary IgAN.

## Supporting information

S1 Table Characteristics of the matched samples. (DOCX)

S2 Table Comparison between patients with secondary IgA according to immunosuppression therapy. (DOCX)
