# Supplementary material for: “Associated” or “Secondary” IgA nephropathy? An outcome analysis
Source: PLoS One. 2019 Aug 9;14(8):e0221014. doi: 10.1371/journal.pone.0221014 (PMC6688810; doi:10.1371/journal.pone.0221014)
Supplement: S1 Table — (DOCX) [file pone.0221014.s001.docx]

**Supporting Information**

|  | **Viral/Autoimmune secondary IgAN** | | | **Liver disease secondary IgAN** | | |
| --- | --- | --- | --- | --- | --- | --- |
|  | **Primary IgAN**  **n=40** | **Secondary IgAN**  **n=40** | **p** | **Primary IgAN**  **n=18** | **Secondary IgAN**  **n=18** | **p** |
| Age (years)  Male gender (%)  Charlson comorbidity index  Obesity (%)  Diabetes mellitus (%)  Hypertension (%)  Serum creatinine (mg/dL)  eGFR (mL/min/1.73m^2^)  Proteinuria g/g creatinine  Haematuria (cells/mm^3^)  Serum albumin (g/dL) | 52 (41.0, 62.0)  87  2.0 (1.0, 4.0)  45  5  86  1.6 (1.1, 2.9)  43.5 (22.9, 69.0)  1.1 (0.6, 1.6)  98 (22, 230)  4.3 (3.9, 4.6) | 53.0 (39.0, 59.0)  69  3.0 (1.0, 5.0)  23  8  69  1.9 (1.3, 2.8)  33.7 (23.1, 62.8)  0.9 (0.4, 1.6)  180 (20, 230)  4.2 (3.8, 4.4) | 0.4  0.06  0.2  0.05  0.6  0.07  0.4  0.5  0.8  0.6  0.1 | 61.5 (53.0, 68.0)  89  3.5 (2.0, 5.0)  44  17  83  2.2 (1.3, 2.9)  28.4 (16.4, 58.0)  1.4 (0.6, 2.6)  37 (12, 95)  4.1 (3.8, 4.6) | 57.0 (50.0, 62.0)  100  5.0 (4.0, 7.0)  28  11  72  2.1 (1.4, 4.5)  29.1 (12.6, 55.0)  1.0 (0.4, 1.9)  280 (130, 1800)  3.7 (3.1, 4.1) | 0.3  0.1  0.06  0.2  0.6  0.4  0.8  0.9  0.4  0.001  0.01 |
| **Renal biopsy (%)**  M1  E1  S1  T1/2  C1/2  MESTC score | 100  24  45  21/8  16/3  2 (1,3) | 90  21  36  21/8  15/3  2 (1, 3) | 0.4  0.7  0.4  0.9  0.9  0.2 | 100  39  61  33/22  22/6  3 (2, 4) | 94  44  67  17/17  17/6  2 (2, 4) | 0.3  0.7  0.7  0.3  0.9  0.4 |
| **Treatment (%)**  IS  RASI | 58  68 | 36  69 | 0.06  0.9 | 44  39 | 56  33 | 0.5  0.7 |
| **Outcome (%)**  Double serum creatinine  ESRD  Kidney end-point (double serum creatinine or ESRD)  Death  Composite end-point (double serum creatinine, ESRD, death) | 3  11  14  5  19 | 8  13  21  13  34 | 0.2  0.7  0.6  0.2  0.2 | 6  22  28  6  34 | 17  6  23  33  56 | 0.5  0.1  0.5  0.03  0.1 |
| eGFR - estimated glomerular filtration rate; ESRD - End stage renal disease; IS - immunosuppression; IgAN - IgA nephropathy; RASI - Renin angiotensin system inhibitors | | | | | | |

**S1 Table. Characteristics of the matched samples**
