# Supplementary material for: “Associated” or “Secondary” IgA nephropathy? An outcome analysis
Source: PLoS One. 2019 Aug 9;14(8):e0221014. doi: 10.1371/journal.pone.0221014 (PMC6688810; doi:10.1371/journal.pone.0221014)
Supplement: S2 Table — (DOCX) [file pone.0221014.s002.docx]

|  | **Immunosuppression** | | **p** |
| --- | --- | --- | --- |
|  | **Yes**  **n=24** | **No**  **n=34** |  |
| Age (years)  Male gender (%)  **Group of disease underlying IgA (%)**   - Autoimmune - Liver disease - Viral infection   Charlson comorbidity index  Obesity (%)  Diabetes mellitus (%)  Hypertension (%)  Serum creatinine (mg/dL)  eGFR (mL/min)  Urinary protein/creatinine  Haematuria (mm^3^)  Serum albumin (g/dL) | 56.5 (49, 60)  83  29  46  25  4 (2, 5)  25  6  71  2.1 (1.1, 3.6)  30.7 (17.5, 63.4)  1.0 (0.7, 1.7)  182 (60, 250)  4.1 (3.7, 4.4) | 54.5 (46, 59)  77  29  29  42  3.5 (2, 5)  26  12  71  1.6 (1.3, 2.4)  43.6 (25.5, 55.0)  0.8 (0.4, 1.5)  200 (89, 325)  4.1 (3.6, 4.3) | 0.8  0.5  0.3  0.7  0.8  0.6  0.9  0.4  0.5  0.2  0.7  0.9 |
| **Renal biopsy findings (%)**  M1  E1  S1  T1/2  C1/2  MESTC score | 92  33  54  38  29  2.5 (2, 4) | 91  24  41  26  15  2 (1, 2) | 0.9  0.4  0.3  0.3  0.1  0.1 |
| **Treatment (%)**  RASI | 50 | 62 | 0.3 |
| **Outcome (%)**  Double sCr  ESRD (dialysis/renal transplantation)  Kidney end-point (double sCr or ESRD)  Death  Composite end-point | 17  17  34  21  55 | 9  24  33  18  51 | 0.3  0.5  0.8  0.7  0.7 |
| RASI renin angiotensin system inhibitor; eGFR, estimated glomerular filtration rate; M1, mesangial hypercellularity; E1, endocapillary hypercellularity; S1, segmental glomerulosclerosis; T1/2, tubular atrophy and interstitial fibrosis >25%; C1/2, crescents in at least one glomerulus; ESRD, end-stage renal disease. | | | |

**Supporting Information**

**S2 Table. Comparison between patients with secondary IgA according to immunosuppression therapy**
